# NEIGHBORHOOD BLUE SPACES AND RISK OF INCIDENT DEMENTIA: IMPORTANCE OF SPATIAL AND HISTORICAL CONTEXT

**DOI:** 10.1093/geroni/igae098.1503

**Published:** 2024-12-31

**Authors:** Kyle Moored, Michael Desjardins, Andrea Rosso, Gina Lovasi, Patrick Donahue, Timothy Shields, Frank Curriero, Michelle Carlson

**Affiliations:** Johns Hopkins Bloomberg School of Public Health, Baltimore, Maryland, United States; Johns Hopkins University, Baltimore, Maryland, United States; University of Pittsburgh, Pittsburgh, Pennsylvania, United States; Drexel University, Philadelphia, Pennsylvania, United States; Johns Hopkins University, Baltimore, Maryland, United States; Johns Hopkins University, Baltimore, Maryland, United States; Johns Hopkins University, Baltimore, Maryland, United States; Johns Hopkins University, Baltimore, Maryland, United States

## Abstract

Neighborhood blue spaces (e.g., lakes, rivers, other water sources) are underexplored contributors to cognitive aging that may act by promoting recreation and mental health. Yet, benefits may vary by historical use and surrounding spatial context of water sources. We examined associations between neighborhood blue spaces and risk of incident dementia in community-dwelling older adults in Pittsburgh, PA. Participants were 655 adults ≥65 years old from the Cardiovascular Health Cognition Study (CHCS; 1992-1999) living in Pittsburgh/Allegheny County, PA. Baseline blue space was measured as the Census tract-level percentage of open water from the 1992 National Land Cover Dataset, stratified into groups incorporating water source: none (0%), low (.003-.04%, smaller water sources), and high (>.04%, major rivers). Time to dementia diagnosis was clinically adjudicated from a neurological exam, neuropsychological examinations, medical records, and proxy reporting across a median 6.2 years (n=103 cases, 16%). Cox models were adjusted for individual age, sex, race, education, income, and lifetime occupation. Greater blue space was associated with higher risk of all-cause dementia (low vs. none: HR = 1.74, 95% CI: 1.26, 2.41 high vs. none: HR = 2.08, 95% CI: 1.45, 2.98). Contrary to our hypothesis, neighborhood blue space was linked to increased risk of dementia for residents of Pittsburgh, PA. Historical industrialization and clustering of lower income neighborhoods along Pittsburgh’s main rivers will be discussed as potential contributors to this finding. Future work will capitalize on the geographic diversity of CHCS by comparing these analyses with more rural/suburban clinic sites (Winston-Salem, NC; and Hagerstown, MD).

